# Accelerating African neuroscience to provide an equitable framework using perspectives from West and Southern Africa

**DOI:** 10.1038/s41467-023-43943-3

**Published:** 2023-12-07

**Authors:** Sahba Besharati, Rufus Akinyemi

**Affiliations:** 1 Department of Psychology, School of Human and Community Development, University of the Witwatersrand, Johannesburg, South Africa; 2 CIFAR Azrieli Global Scholars Program, CIFAR, Toronto, Canada; 3 Neuroscience and Ageing Research Unit, Institute for Advanced Medical Research and Training, College of Medicine, University of Ibadan, Ibadan, Nigeria; 4 Centre for Genomic and Precision Medicine, College of Medicine, University of Ibadan, Ibadan, Nigeria

**Keywords:** Medical research, Scientific community, Cognitive neuroscience, Developing world

## Abstract

Drawing on perspectives from West and Southern Africa, this Comment critically examines the current state of neuroscience progress in Africa, describing the unique landscape and ongoing challenges as embedded within wider socio-political realities. Distinct research opportunities in the African context are explored to include genetic and bio-diversity, multilingual and multicultural populations, life-course development, clinical neuroscience and neuropsychology, with applications to machine learning models, in light of complex post-colonial legacies that often impede research progress. Key determinants needed to accelerate African neuroscience are then discussed, as well as cautionary underpinnings that together create an equitable neuroscience framework.

The rising discourse around equity, diversity and inclusion in science has propelled international focus on the current landscape of neuroscience in Africa. Neuroscience has its origins in Africa, with ancient Egypt boasting the first anatomical investigations of the brain^1^. In more recent decades, neuroscience - as an interdisciplinary field - has witnessed remarkable growth with the rise in the neuroimaging and computational sciences, driven by rapid technological advances. Such progress is yet to be achieved in arguably less research intensive and economically powered countries across the African continent. Despite the need to steer away from broad over-generalisations, many of the challenges and prospects outlined here may be echoed in what has been referred to as countries in the Global South, low-to-middle income countries or Majority World, and in some cases, to underrepresented populations across the globe.

## Historical and current landscape of neuroscience progress in Africa

As skillfully mapped out by Maina and colleagues’ review^2^ of two decades of neuroscience research from the continent, there is a steady and encouraging rise in publications, dedicated neuroscience centres and funding prospects. This upward trajectory is most evident in specific regions and countries in North (Morocco, Tunisia and Egypt), West (Nigeria) and Southern Africa (South Africa), with notable publication increases in East (e.g., Kenya) and central African countries. A growth in neuroscience outputs by smaller and less populated African countries and islands is particularly noteworthy. This rising trend however has been made against the backdrop of a post-colonial environment often characterised by political unrest, violence, poor basic infrastructure, and economic hardship. To conduct a critical analysis of the current state and future potential for growth, it is necessary to assume a historical lens and unpack the consequences of the wider socio-political climate.

Using South Africa as an example, there is a strong research tradition of the neurosciences in behavioural and clinical disciplines spread across academic centres throughout the country. Cognitive science (e.g., psychometric testing) and neuroimaging methods like electrophysiological (EEG) were utilised very early on as aligned with more international trends^3^. However, such methods were used by the previous apartheid government to try and produce “scientific” evidence to justify highly racist legislative policies^4^, with local research funding often dependent on alignment with discriminatory government policy^5^. When findings were not aligned to the government agenda, as in the case with EEG data showing no cross-cultural difference in responses, researchers were heavily criticised and government research funding restricted^6,7^. As such, while there was rapid growth in pioneering neuroimaging technologies in the Global North, the highly politicised nature of research and research funding restricted local scientific progress^8^. Years later in 2007, after the political transition in South Africa, the Cape Universities Body (previously Brain) Imaging Centre (CUBIC) was established, housing the first dedicated research magnetic resonance imaging (MRI) scanner in Africa^9^.

**Fig. 1:**
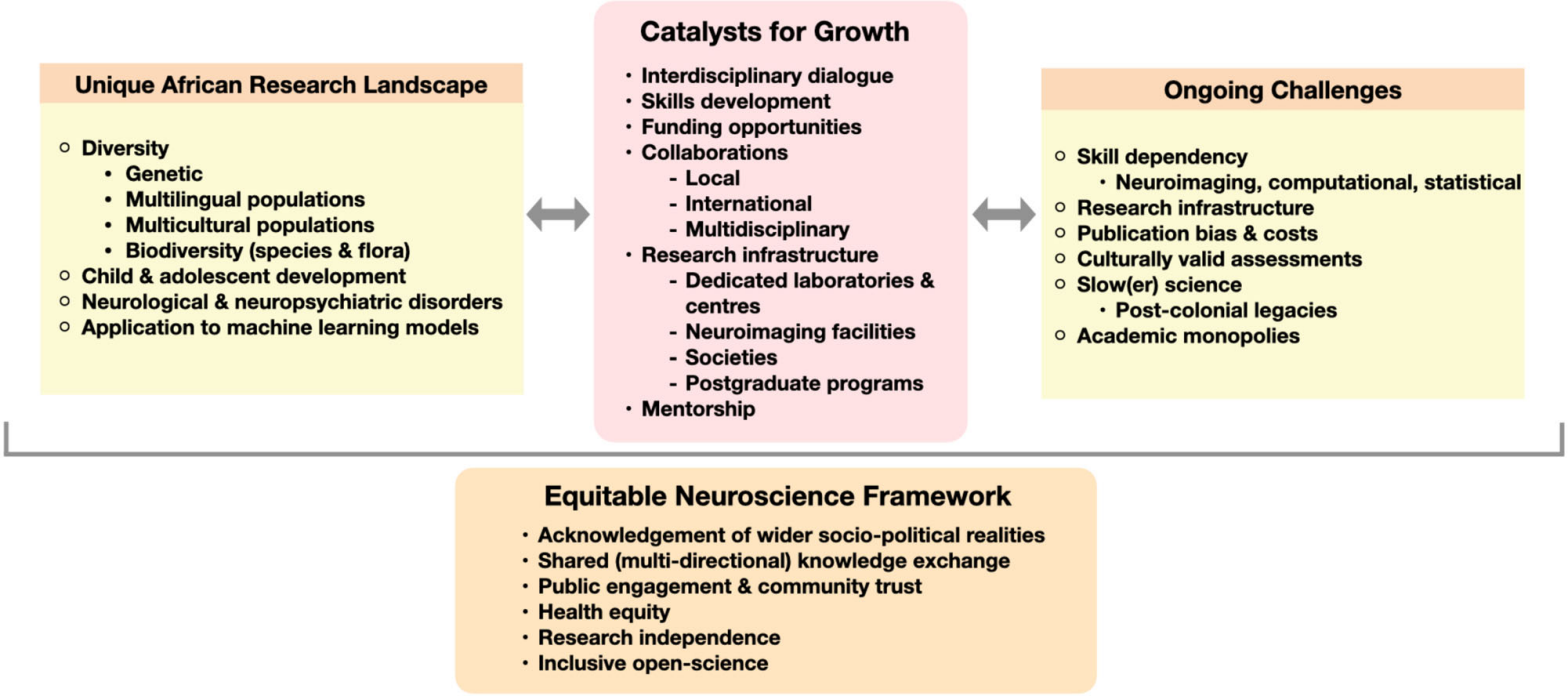
Re-framing African neuroscience. This graphical depiction provides a synthesised account of the unique research opportunities for neuroscience in Africa, together with ongoing research challenges that can be counteracted by identified key markers for growth. Within this context, a new equitable neuroscience framework is proposed that has applications for the global neuroscience community.

Despite this post-colonial backdrop, neuroscience research in Africa provides a unique research landscape that allows for the investigation of research questions and areas that are not always available in other parts of the world^10^ (see Fig. 1). In view of the wider socio-political environment in Africa, the prevalence of neurological disorders, such as stroke and traumatic brain injury, are among the highest in the world in countries like Nigeria and South Africa^11,12^. The high burden of disease, paired with unique genetic polymorphisms, underscore both the necessity and distinct contributions of the clinical neurosciences. Africa—“as the cradle of the mankind”—houses the African genome that demonstrates the highest diversity globally, which has far-reaching potential to study neurological diseases^13^. Similarly, the enormous biodiversity in Africa has far reaching potential. The diversity in African flora presents opportunities for novel advances in neuropharmacology^14^ for example, while comparative neuroscience research has the advantage of a wonderful array of species diversity^15^.

Furthermore, understanding brain development over the life course has a unique place in Africa. An upward population growth trajectory in Africa^16^ affords many avenues to investigate cognitive and brain development from early childhood development to adolescence in both healthy and clinical populations. The use of population-based and birth cohorts in Africa^17,18^ is one example of using multidisciplinary data to look at longitudinal perspectives of multiple psychosocial and neurophysiological outcomes as applied to health and education equity.

However, the distinct research potential for the neurosciences in Africa is not only in health-related research. The rich cultural and linguistic diversity of the continent lends itself to the cognitive and social neurosciences. Highly multilingual populations in Africa for example, can offer a fresh perspective on the ongoing debate of the bilingual advantage^19^. Including multicultural populations in research not only enhances the validity and generalisability of cognitive phenomena, but also introduces important social dimensions to neuroscience research. Recent progressive calls towards non-‘WEIRD’ (western, educated, industrialised, rich and democratic)^20^ science, for example, excludes a conversation on race by omitting the bias in using majority white population groups^21^. Including important cross-cultural comparisons in social-cognitive neuroscience extends the use of the catchphrase ‘WEIRD’ to include biases in *white and* western populations. There is also a pressing need to increase research outputs with culturally diverse samples into training corpora for machine learning algorithms to counteract implicit biases and stereotyping with the recent surge in artificial intelligence systems and large language models^22^.

## On-going challenges

Despite this unique potential for neuroscience innovation, the wider socio-political climate in Africa (e.g., political unrest and student protests, continued power-shortages, government research funding, violence, discriminatory practices) have a direct and indirect impact on research progress^23^. This ultimately leads to slow(er) science. However, African neuroscientists and trainees are still evaluated using competitive international matrices for research progress (e.g., h-index, or amount of funding received). Continued infrastructural challenges, including a lack of dedicated research laboratories and neuroimaging facilities, also stunt innovation potential. Although it is encouraging to see a growth of dedicated centres for neuroscience research in recent years, having them condensed to one city or university, creates ‘academic monopolies’. Mirroring the economic organisation of many African countries, this saturation of neuroscience research in one academic setting often limits opportunities for students, employment and its application of research to the wider community.

In places where there is access to sophisticated research methods, there is still a skill dependency for analysis of data. For example, the use of MRI methods is increasing in cognitive and clinical neuroscience research. Pioneering efforts are being made to increase local capacity in MRI methods^24^ specifically and in broader neuroscience skills^25^. Nevertheless, the analysis of MRI data, as an example, is still mostly outsourced to collaborators in the Global North rather than targeting local capacity development. The shortage of validated and culturally appropriate cognitive and affective assessment tools remains a challenge. This challenge is then compounded by limited normative data for culturally and linguistically diverse populations coming from a range of educational and socio-economic backgrounds^26^. Publication bias already well documented for women and underrepresented groups are echoed for African neuroscientists^27^, with inaccessible costs for open-access publications for top-tier journals further limiting impact potential. These inequities even filter down to the use of experimental equipment, with caps for EEG research for example often not being suitable for diverse hair types^28^.

The scope for local research funding can be limited, which leads to inadequately powered studies that impends research impact. Many scientists often resort to using personal savings to support research, with few principal investigators having funds to support graduate students. PhD students are often self-funded, with limited opportunities for Postdoctoral Fellowships. Taken together, poor renumeration, work insecurity, limited training opportunities and funding are often push factors for rapid emigration of skilled African researchers^29^.

## Catalysts for change embedded within an equitable neuroscience framework

Surrounded by the research obstacles only briefly outlined here, African neuroscientists have been resourceful and inventive to produce impactful work. Key ingredients can be identified to catalyse growth potential. Despite challenges, the funding landscape for neuroscience research in Africa is growing. Local, African specific research investments in the last decade have propelled research progress on multiple fronts. For example, the Human Health and Heredity (H3Africa) and Data Science (DSI – Africa) initiatives have boosted African participation in the genomic revolution, built genomic research infrastructure and enhanced training of young African scientists as well as localising the analysis of big data on African soil^30^. International funding and partnerships with large organisations like the National Institute of Health in the USA and the UK’s Wellcome Trust have also been transformative for research groups.

Access to more sophisticated research techniques also needs to run in parallel with local skill development. Strengthening research capacity through visiting placements, student exchanges, collaborative or shared graduate trainees, targeted training workshops, utilising virtual or hybrid methods, have all proven to be sustainable strategies to build critical skills and foster research independence. Targeted mentorship of graduate trainees and postdoctoral fellows also sharpens this impact. Similarly, building local, international and multidisciplinary collaborations is another critical factor to facilitate knowledge exchange between countries and disciplines. However, a cautionary note is needed here that international collaborations and initiatives for “capacity building” are not unidirectional and do not replicate historic power hierarchies of the knowledgeable and uninformed. Rather a shared and multidirectional knowledge exchange is needed. In a similar respect, collaborative research initiatives should encourage more African principal investigators, with equal ownership of data and credit for arising publications.

Societies and platforms for networking and building interdisciplinary spaces have been an effective strategy for growth and vision building. Established organisations like the Society of Neuroscience of Africa (SONA), the Southern African Neuroscience Society (SANS) and the International Brain Research Organisation (IBRO) have been widely effective. More recent initiatives are also gaining traction, like the African neuroscience ALLIANCE, clinical consortia such as the African Dementia Consortium (AfDC)^31^ or the African Stroke Organization (ASO)^32^, and international non-profit organisations such as TReND in Africa and the Neuroscience Alliance (NEURAL) that promote global engagement and healthy equity. In a similar respect, effective science communication initiatives have had encouraging results in building community trust and awareness, as well as in supporting science-informed policy change^33^.

## Concluding remarks

What remains pivotal is that *who* does science is important. Having greater representation in neuroscience research from Africa affords unique opportunities for new research trajectories to push forward our understanding of the brain. Purposeful and directed action is needed by multiple stakeholders to move towards this goal.
